# Genome‐resolved metagenomics and metatranscriptomics reveal niche differentiation in functionally redundant microbial communities at deep‐sea hydrothermal vents

**DOI:** 10.1111/1462-2920.14806

**Published:** 2019-10-17

**Authors:** David Galambos, Rika E. Anderson, Julie Reveillaud, Julie A. Huber

**Affiliations:** ^1^ Biology Department Carleton College Northfield Minnesota USA; ^2^ INRA Animal Health Department 37380 Nouzilly France; ^3^ Marine Chemistry and Geochemistry, Woods Hole Oceanographic Institution Woods Hole Massachusetts USA

## Abstract

The structure and function of microbial communities inhabiting the subseafloor near hydrothermal systems are influenced by fluid geochemistry, geologic setting and fluid flux between vent sites, as well as biological interactions. Here, we used genome‐resolved metagenomics and metatranscriptomics to examine patterns of gene abundance and expression and assess potential niche differentiation in microbial communities in venting fluids from hydrothermal vent sites at the Mid‐Cayman Rise. We observed similar patterns in gene and transcript abundance between two geochemically distinct vent fields at the community level but found that each vent site harbours a distinct microbial community with differing transcript abundances for individual microbial populations. Through an analysis of metabolic pathways in 64 metagenome‐assembled genomes (MAGs), we show that MAG transcript abundance can be tied to differences in metabolic pathways and to potential metabolic interactions between microbial populations, allowing for niche‐partitioning and divergence in both population distribution and activity. Our results illustrate that most microbial populations have a restricted distribution within the seafloor, and that the activity of those microbial populations is tied to both genome content and abiotic factors.

## Introduction

First discovered in 1977, deep‐sea hydrothermal vents host flourishing ecosystems that are fuelled by chemosynthetic microbes. Diffuse fluids emerging from the seafloor near hydrothermal vents represent a mix of hydrothermal fluid and deep seawater and provide a window into the subseafloor microbial habitat (Jannasch and Motti, 1980). The mixing of cold background seawater with high‐temperature hydrothermal fluid creates gradients in temperature, pH, and energy sources, supporting diverse microbial communities with population structures that vary depending on the local geological and geochemical environment (Takai and Horikoshi, 1999; Huber *et al*., 2007; Akerman *et al*., 2013; Perner *et al*., 2013; Anderson *et al*., 2015; Meier *et al*., 2017).

Differences in microbial community composition become particularly clear when comparing mafic systems—which are characterized by high temperature acidic fluids enriched in sulphide and other metals—with ultramafic systems, which are characterized by relatively cooler basic fluids enriched in hydrogen, methane, and other hydrocarbons (Amend *et al*., 2011). Ultramafic systems are often inhabited by methane and hydrogen metabolizing bacteria and archaea (Brazelton *et al*., 2006, 2010; Flores *et al*., 2011; Perner *et al*., 2013). In contrast, these microbial groups occur in lower abundance in mafic systems, which tend to be dominated by sulphur‐oxidizing bacteria (Huber *et al*., 2007; Akerman *et al*., 2013; Anderson *et al*., 2013; Meier *et al*., 2017; Trembath‐Reichert *et al*., 2019). Despite commonly observed differences in microbial community structure at various hydrothermal systems in distinct geological settings, the functional repertoire of microbial communities is sometimes more similar across hydrothermal systems than their taxonomic composition might imply. This was recently observed at the Mid‐Cayman Rise, located on an ultraslow spreading ridge in the Caribbean Sea, where two distinct types of vent systems exist: the mafic Piccard vent field, which is the deepest vent site discovered to date at a depth of 4960 m, and the ultramafic Von Damm vent field, located 20 km away on top of a massif at 2350 m depth (German *et al*., 2010). Using 16S rRNA gene sequencing and metagenomics, Reveillaud *et al*. (2016) examined diffuse fluids from both Von Damm and Piccard and showed that each vent field hosts phylogenetically distinct microbial communities. However, metagenomic analyses of one sample from each site showed that although a wider diversity of metabolisms was observed at Von Damm, including anaerobic methane oxidation, the microbial communities at the two vent fields had near functional equivalence, with metabolisms related to methane, hydrogen, and sulphur cycling. The authors hypothesized that these similarities in functional repertoire likely result from the high concentrations of both hydrogen and sulphide available at both sites (McDermott *et al*., 2015, 2018; Reveillaud *et al*., 2016).

Louca *et al*. (2018) suggested that functional redundancy at the metabolic gene level may mask niche differentiation manifested in other parts of the genome, resulting from biotic interactions like viral infection. Accordingly, previous work examining metagenome‐assembled genomes (MAGs) from Von Damm and Piccard vent fluids showed that differential selection pressure has favoured different strains within the same taxa at Von Damm and Piccard (Anderson *et al*., 2017). These analyses showed that microevolutionary processes diverged between the Piccard and Von Damm vent fields, as different populations showed evidence of clonal expansions or selective sweeps in each site. However, it is also possible that differences might emerge at the level of gene expression, such that the fundamental niche (metabolic potential based on DNA) is distinct from the realized niche (expression of genes based on messenger RNA) for microbial populations with similar metabolic repertoires. Here, we combine metagenomic and metatranscriptomic data with MAGs to assess potential niche differentiation at the levels of genes, transcripts, and genomes in microbial communities from venting fluids of the Mid‐Cayman Rise. We compared metabolic potential and transcript abundance at both the individual gene and genomic level across multiple vent sites over two years and analysed metabolic pathways in MAGs to search for niche differentiation at higher genomic resolution. We show that although Von Damm and Piccard are distinguished by a few distinct patterns in overall transcript abundance, clear differences emerge at the level of individual populations within each vent field.

## Results

We examined 15 metagenomes and 10 metatranscriptomes from 12 different diffuse flow vent sites at the Von Damm and Piccard vent fields on the Mid‐Cayman Rise to reconstruct metabolic potential, transcript abundance and population dynamics in subseafloor microbial communities of two geologically distinct deep‐sea hydrothermal systems (Table 1). These low‐temperature diffuse vent samples represent mixtures of hot hydrothermal vent fluid and deep seawater that mix and react beneath the seafloor. We sampled fluids over a 30–40 min time period and saw natural fluctuations in temperature during that sampling period, with the maximum temperature recorded in Table 1 (Reveillaud *et al*., 2016). A detailed description of the samples, their geochemistry, cell abundances, and sequencing statistics can be found in Supporting Information Table S1 and in Reveillaud *et al*., 2016 and Anderson *et al*., 2017. At both Piccard and Von Damm, previous geochemical analyses of the vent fluids by McDermott *et al*. (2015, 2018) showed that each vent field hosts a single deep‐rooted source fluid that feeds all of the individual diffuse vent sites. This means that all of the diffuse fluids sampled from Von Damm result from the mixing of the same high temperature source fluid with seawater beneath and at the seafloor (McDermott *et al*., 2015), and a similar process occurs at Piccard with a single source fluid feeding each individual site at that vent field (McDermott *et al*., 2018).

**Table 1:** Data regarding samples included in this study.

Sample name	Sample number	Vent field	Year	T_Max_ (°C)	MG/MT
Old Man Tree	FS841	Von Damm	2012	114	MG & MT
Ravelin #2	FS842	Von Damm	2012	86	MG only
Shrimp Hole	FS844	Von Damm	2012	50	MG & MT
Ginger Castle	FS848	Von Damm	2012	47	MG & MT
Main Orifice	FS849	Von Damm	2012	109	MG only
Hot Chimlet, BVM	FS851	Piccard	2012	106	MG & MT
Shrimp Canyon, BVM	FS852	Piccard	2012	44	MG & MT
X‐19 at BV #4, BVM	FS854	Piccard	2012	18	MG & MT
Shrimp Gulley #2, BSM	FS856	Piccard	2012	108	MG & MT
Near Main Orifice	FS866	Von Damm	2013	130	MG only
Shrimp Hole	FS872	Von Damm	2013	30	MG & MT
Twin Peaks	FS874	Von Damm	2013	140	MG only
Shrimp Buttery	FS877	Von Damm	2013	131	MG only
Hot Cracks #2	FS879	Von Damm	2013	29	MG & MT
Old Man Tree	FS881	Von Damm	2013	114	MG & MT

The MG/MT indicates whether both a metagenome (MG) and a metatranscriptomes (MT) were sequenced for that sample, or a metagenome only. Supporting Information Table S1 provides additional data for each sample.

### *Metabolic gene abundance and transcript abundance across samples*

**Figure 1:**
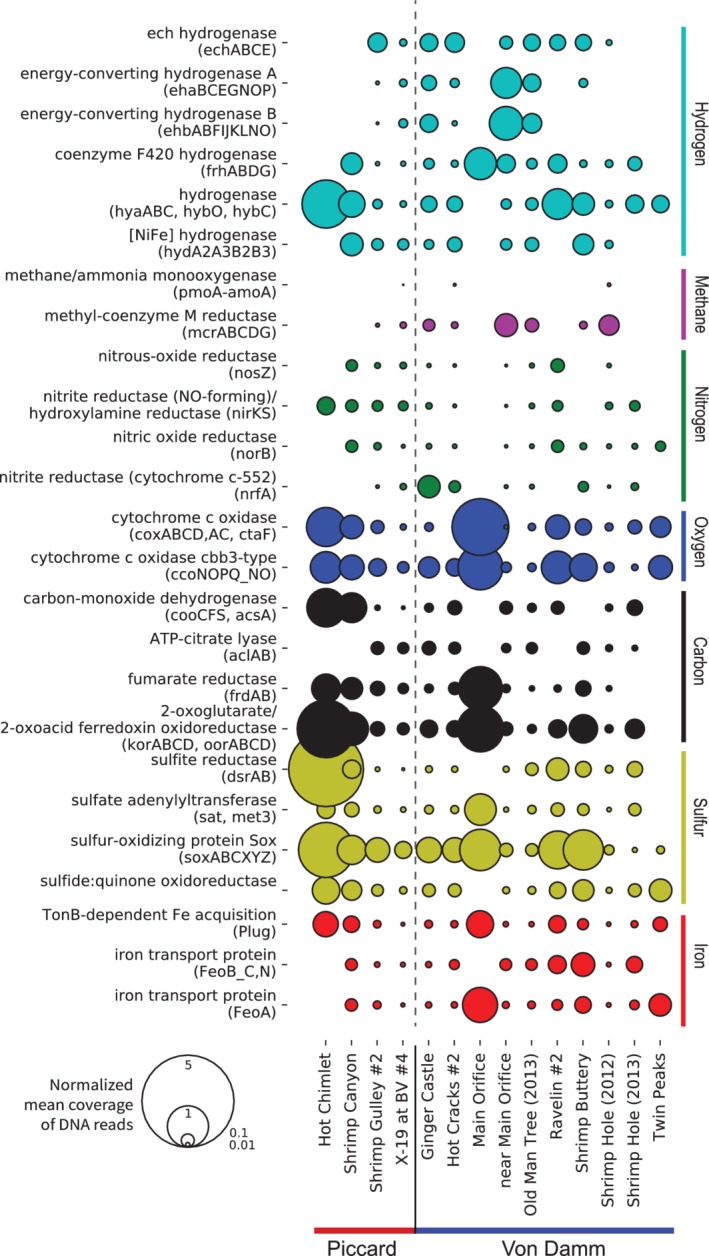
Relative abundance of key metabolic genes in metagenomic samples from the Von Damm and Piccard vent fields. Bubble size represents the normalized mean coverage of a specific gene type based on metagenomic mapping. We calculated the normalized gene abundance for each sample by dividing the number of metagenomic read hits to each key gene by the average number of metagenomic read hits to 35 single copy COGs.

To ascertain functional potential at the community level across different vent sites, we examined the relative abundance of key metabolic genes across all 15 metagenomes. Metabolic genes were chosen from specific pathways of interest, including sulphur, oxygen, nitrogen, hydrogen, iron, and methane metabolism (Reveillaud *et al*., 2016) (Fig. 1). The overall patterns in functional potential were similar between the Von Damm and Piccard vent fields at the community level. A few differences emerged: for example, microbial communities at the vent sites Main Orifice (Von Damm) and Hot Chimlet (Piccard) both had much higher abundances of cytochrome c oxidases, 2‐oxoacid ferredoxin oxidoreductase and sulphur‐oxidizing proteins (Sox) compared to other vents, although Main Orifice was completely lacking sulphite reductase and hydrogenase in comparison to most other vents (Fig. 1). Overall, however, we did not observe clear distinctions between the Von Damm and Piccard vent fields (Fig. 1). Some genes were universally present across all sites sampled, including hydrogenase, cytochrome c oxidase, sox genes, and the TonB‐dependent iron acquisition genes (Fig. 1). This is consistent with previous results using one metagenome each from Piccard and Von Damm (Reveillaud *et al*., 2016).

**Figure 2:**
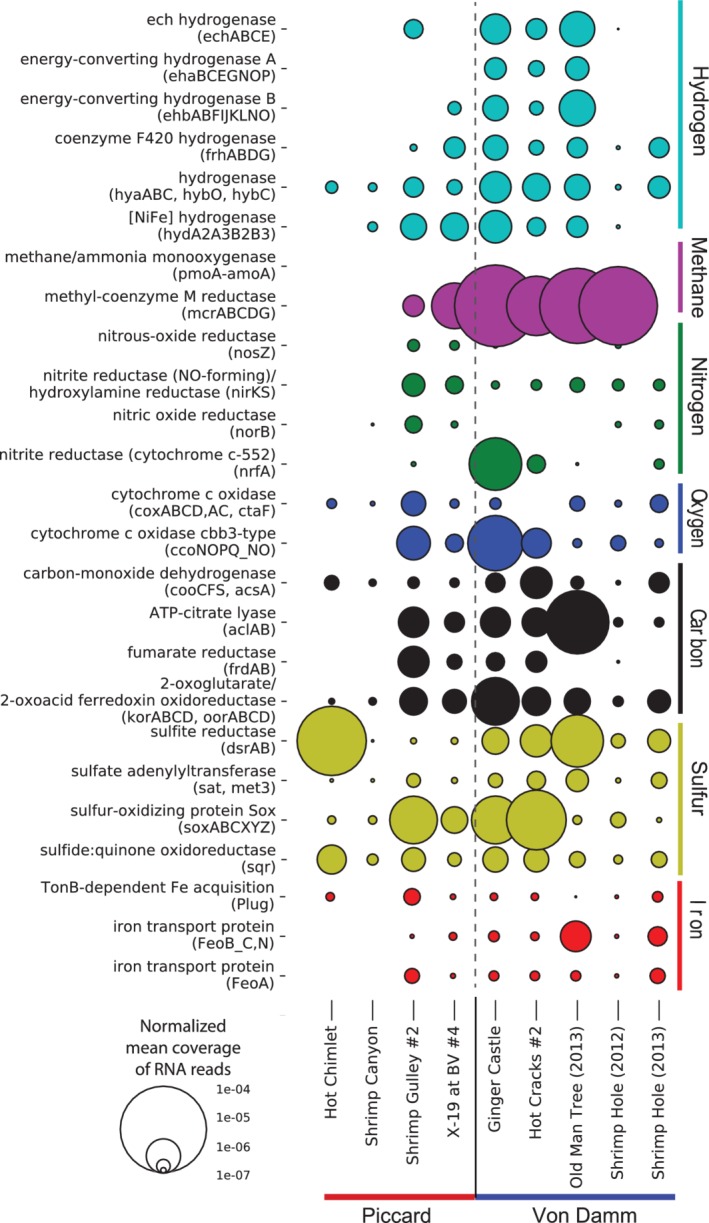
Relative transcript abundance of key metabolic genes in metatranscriptomic samples from the Von Damm and Piccard vent fields. Genes are categorized according to the compounds they metabolize. Bubble size represents the normalized mean coverage of a specific gene type based on metatranscriptomic mapping. To calculate transcript abundance of each gene, we divided the number of metatranscriptomic read hits to each key gene by the total number of metatranscriptomic reads.

In addition to community‐level gene abundance, we also examined community‐level transcript abundance by mapping metatranscriptomic reads to key metabolic genes. Although there were no large‐scale patterns differentiating Piccard and Von Damm, some genes showed different patterns in transcript abundance between Von Damm and Piccard (Fig. 2). Compared to Piccard, samples from Von Damm displayed higher transcription of some hydrogenases and the methanogenesis gene methyl co‐enzyme M reductase (*mcr*). In addition, the Piccard sites Hot Chimlet and Shrimp Canyon, with few exceptions, had extremely low transcript abundance for all genes compared to the other vent fluids. Overall, we observed that a wider set of genes was expressed at the Von Damm sites Ginger Castle, Hot Cracks and Old Man Tree compared to vent sites at Piccard (Fig. 2).

### *MAG‐resolved transcript abundance across samples*

Seventy three MAGs with >70% completeness and <10% redundancy from 15 metagenomes were collected at the Piccard and Von Damm vent fields at the Mid‐Cayman Rise by Anderson *et al*. (2017) (Supporting Information Table S2). Here, all *Pseudomonadales* and *Sphingomonadales* MAGs (9 of 73 total MAGs) were excluded from additional analysis, as they are most likely seawater organisms mixing with vent fluids at the point of sampling and are thus unlikely to be relevant subseafloor and vent microorganisms.

**Figure 3:**
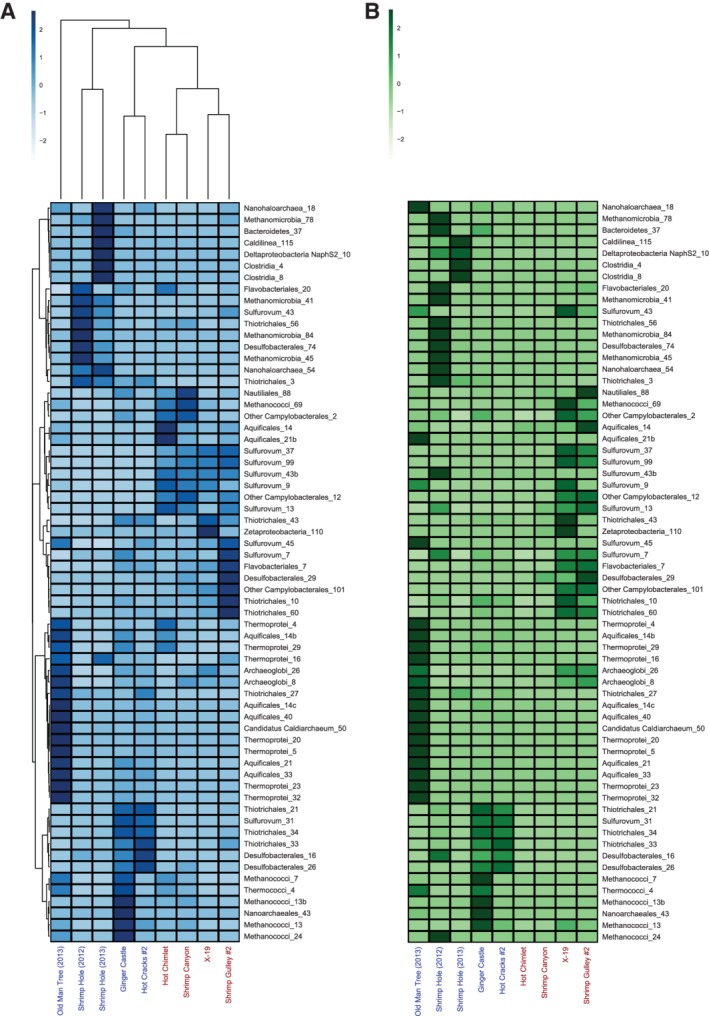
Normalized abundance of metagenomic and metatranscriptomic read mapping for MAGs across samples. For both heatmaps, the *x*‐axis shows samples from Piccard (red) and Von Damm (blue). A. Heatmap of MAG transcript abundance across samples. MAG metatranscriptomic coverage values were normalized using the number of metranscriptomic reads in each sample. B. Heatmap of MAG metagenomic read abundance across samples. MAG metagenomic coverage values were normalized using the number of metagenomic reads in each sample. For both heatmaps: x‐axis shows samples at Piccard and Von Damm; y‐axis shows high‐quality MAGs. A z‐score transformation was applied to each row; the legend indicates the z‐score for a cell relative to the mean for all values in that row. A MAG has an elevated z‐score if its coverage at that site is higher than the average for the MAG across all sites. Dendrograms in part (A) indicate hierarchical clustering for MAGs and samples based on z‐scored values according to the metatranscriptomic coverage. The samples and MAGs in part (B) were ordered to match the clustering order shown in part (A).

To determine whether there were any patterns in microbial activity distinguishing Von Damm and Piccard at the population level, we performed a hierarchical clustering of both the MAGs and samples based on their respective patterns of transcript abundance (Fig. 3A). All four Piccard sites clustered together, while the Von Damm sites were less similar to one another, with the two samples from Shrimp Hole clustering together, and Ginger Castle and Hot Cracks #2 grouping with one another (Fig. 3A). None of the MAGs displayed uniform transcript abundance across all samples (Fig. 3A). There were several *Sulfurovum* MAGs that showed elevated transcript abundance at all four Piccard sites, but most MAGs showed expression at a single Von Damm vent. The two samples from Shrimp Hole hosted a distinct group of MAGs, including those belonging to Methanomicrobiales and Deltaproteobacteria, whereas the other Von Damm vents (Old Man Tree, Ginger Castle, and Hot Cracks) were dominated by high‐temperature bacteria and archaea including the Desulfobacteriales, Archaeoglobales, Thermococcales, and Methanococcales (Fig. 3A). Different MAGs belonging to bacterial groups Aquificales, *Sulfurovum* and Thiotrichales were found at both Von Damm and Piccard.

To determine the extent to which the relative abundance of a MAG correlated with its transcript abundance, we compared the coverage of metagenomic and metatranscriptomic reads within MAGs (Fig. 3B). In some cases, population abundance matched population transcript abundance. For example, many MAGs were only found at high abundance at Old Man Tree in 2013 (Fig. 3B), and the same MAGs were highly expressed at that site (Fig. 3A). A similar pattern occurred for MAGs that were both highly abundant and highly expressed at Ginger Castle and Hot Cracks #2. However, in other cases, MAG abundance and transcript abundance did not correlate. For example, although *Sulfurovum* MAGs were usually only found at high abundance in a single vent site (such as at X‐19 or Shrimp Gulley #2), many *Sulfurovum* MAGs were expressed at moderately high levels across all four vent sites at Piccard vent field (Fig. 3A and B).

### *Functional potential of microbial lineages via metabolic pathways*

We assessed the functional potential of each MAG by defining a module completion score (MCS) for each MAG that describes the functional capability of a MAG while allowing for the existence of multiple enzyme combinations for any step of the pathway, each of which may be sufficient to complete the pathway. This is based on (but not identical to) the module completion ratio (MCR) calculated by Takami *et al*. (2012). An MCS score of 1 indicates a complete pathway was present in an individual MAG, whereas an MCS score of 0 indicates the pathway was not present in the MAG (code deposited at https://github.com/carleton-spacehogs/functional_metagenomics). For this analysis, we selected 56 KEGG modules related to microbial metabolic pathways, including carbohydrate metabolism, carbon fixation, methanogenesis and aerobic methane oxidation, sulphur and nitrogen reduction and oxidation, and various membrane proteins such as cytochromes and Mn/Zn/Fe/S/N transporters. Supporting Information Table S3 shows the KEGG module accession numbers associated with each pathway.

**Figure 4:**
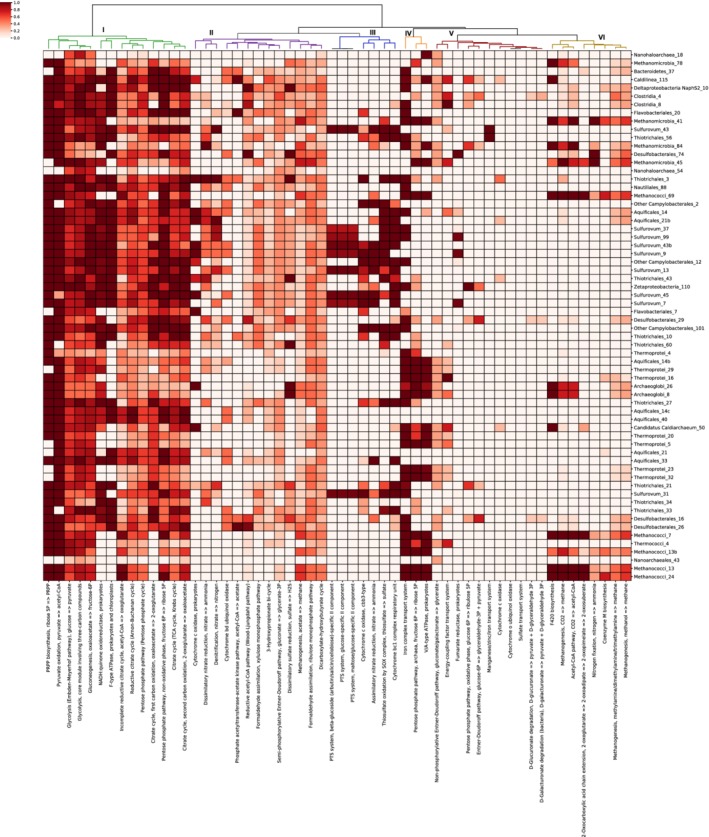
Metabolic potential of MAGs for key metabolic modules. The *x*‐axis shows metabolic pathways selected from the KEGG modules database; the *y*‐axis shows high‐quality MAGs. The legend shows the module completion score for each MAG in each module. Module completion was based on the presence or absence of a given KEGG annotation in all the contigs of a particular MAG. Dendrograms show hierarchical clustering of metabolic pathways based on MCSs. MAGs are ordered to match the metatranscriptomic coverage clustering order shown in Fig. 3. The colouring of the branches represents the six clusters discussed in the text.

Through module completion analysis, six distinct metabolic clusters emerged (Fig. 4). The MAG ordering shown in Fig. 3 is preserved in Fig. 4, allowing for comparison of metabolic potential and transcript abundance patterns for MAGs across sample sites. The first cluster (I) consisted of core carbon cycle metabolisms that were highly conserved in most MAGs with MCS > 0.8. This included modules related to glycolysis, gluconeogenesis, the TCA cycle, and a generic pentose phosphate pathway. Cluster II was highly conserved across the majority of the MAGs, but most of the MCS values were only about 0.5. This cluster also included pathways such as the Wood‐Ljungdahl pathway, denitrification, dissimilatory nitrate reduction and dissimilatory sulphate reduction with high (>0.9) MCS in 2–6 MAGs each. For example, the Wood‐Ljungdahl pathway had a high MCS score in *Desulfobacterales* MAGs, denitrification had a high MCS score in *Aquificales* and *Sulfurovum* MAGs, and dissimilatory nitrate and sulphate reduction had high MCS scores in *Thiotrichales* MAGs. Cluster III consisted of a set of pathways related to thiosulfate oxidation, assimilatory nitrate reduction, phosphate transport system (PTS) sugar uptake, and cytochrome c oxidase where MAGs mostly show MCS≥0.9 or MCS≤0.3. Many of these pathways had high MCS scores in several *Sulfurovum* and other Campylobacterial MAGs. Cluster IV contained many pathways that were highly complete in several archaeal MAGs, including methanogens, Archaeoglobi and Thermoprotei. Cluster V consisted of a set of rare pathways that were only present in a few MAGs with MCS > 0, including the fumarate reductase pathway and the oxidative phase of the pentose phosphate pathway. However, a number of pathways from this group, such as the SO_4_^2−^ and Mg/Zn/Fe transport system, were present with high MCS scores in 2–4 MAGs each. The next cluster (VI) contained modules mostly related to methane metabolism. The most prominent lineages with capabilities in this cluster included *Methanomicrobia* and *Methanocci,* and Archaeoglobi contained many genes in these pathways as well. We observed three methanogenesis modules in the cluster along with pathways for F420 biosynthesis, coenzyme M biosynthesis, acetyl‐CoA to CO_2_ and nitrogen fixation.

In order to search for potential metabolic interactions between specific microbial populations, we compared MAG transcript abundance patterns to the metabolic modules found in each MAG. If specific microbial populations appeared to be active in the same samples, we compared the completion of metabolic pathways across coexpressed MAGs to identify complementary genes in shared metabolic pathways, or 'cross‐feeding' of metabolic pathways, which may reflect metabolic interactions. Two *Nanohaloarchaea* and *Nanoarchaea* MAGs displayed low MCS for all pathways except those involving gluconeogenesis and glycolysis (Fig. 4), and these MAGs had the highest transcript abundance at three separate sites, with two of them having transcript abundance patterns that were tightly correlated with another MAG, as evidenced by clustering patterns (Fig. 3A): Nanoarchaea_43 was active at Ginger Castle and was coexpressed with Methanococci_13b, Methanococci_13 and Methanococci_24, while Nanohaloarchaea_54 and Thiotrichales_3 were both active at Shrimp Hole in both years of sampling (Fig. 3A), suggesting a possible metabolic syntrophy for these lineages.

Given these correlations, we tested whether certain pathways were enriched in groups of MAGs with similar transcript abundance patterns across vent sites (Supporting Information Tables S4‐S6). Overall, most *Sulfurovum* MAGs in the analysis were expressed at Piccard, and the pathway for thiosulfate oxidation was significantly enriched (*p* < 1E−04) in these MAGs compared to MAGs that were expressed elsewhere. The associated pathways for assimilatory nitrate reduction (*p* < 0.01), the cytochrome bc1 complex (*p* < 0.005) and cytochrome c oxidase cbb3 (*p* < 0.05) were significantly enriched as well. In addition, there were several pathways such as the non‐oxidative phase of the pentose phosphate pathway and prokaryotic NADH quinone oxidoreductase that were significantly enriched at Piccard with average MCS > 0.85.

**Figure 5:**
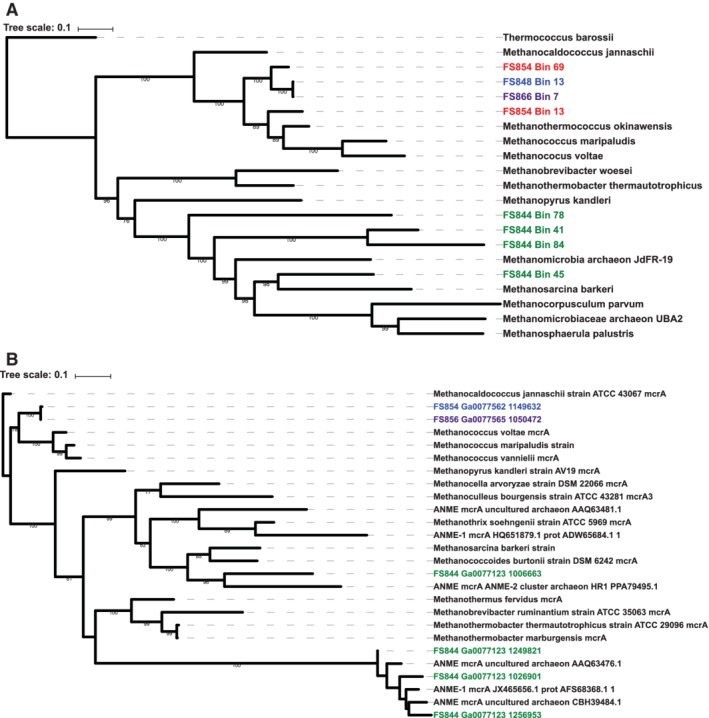
A. Phylogenetic tree of bins classified as methanogens recovered from Piccard and Von Damm. Bins from Von Damm are coloured in blue or purple (depending on sample), Piccard in red, Shrimp Hole in green. Single‐copy genes were identified and aligned using PhyloSift; tree was created using RAxML. B. Phylogenetic tree of mcrA genes identified in metagenomes from Piccard and Von Damm. Genes from samples at Piccard are coloured blue and purple (depending on sample); genes from samples at Shrimp Hole are coloured green. Genes were identified using KEGG annotations and aligned with MUSCLE, and the tree was created using RAxML.

In line with these data, we also observed differences in how certain pathways were distributed among the MAGs. Although the *Methanococci* and *Methanomicrobia* MAGs showed similar metabolic capabilities, the *Methanomicrobia* were active exclusively at Shrimp Hole, while *Methanococci* were active at Shrimp Gulley, Shrimp Canyon, Ginger Castle and Hot Chimlet (Fig. 3A). Because the same set of genes are involved in both methanogenesis and anaerobic oxidation of methane (ANME) pathways, they are difficult to distinguish at the module level. To determine whether the high MCS values for methanogenesis modules seen in the *Methanococci* and *Methanomicrobia* MAGs were associated with methanogenesis or ANME, we created a phylogenetic tree of these MAGs that included known ANME genomes as well as a tree of the *mcrA* genes from these MAGs (Fig. 5). At Shrimp Hole, the mcrA genes found in the *Methanomicrobia* MAGs were most closely related to mcrA genes with known ANME function. In addition, the *Methanomicrobia* MAGs were most similar to previously identified *Methanomicrobia* and *Methanosarcina*. In contrast, mcrA sequences from sites other than Shrimp Hole clustered with mcrA genes from known methanogens, and the *Methanococcus* MAGs clustered with methanogens, including other *Methanococci*.

## Discussion

In this study, we used metagenomic and metatranscriptomic data of microbial communities in venting fluids from the Mid‐Cayman Rise to examine functional potential and activity at the gene‐ and genome‐resolved scale in the subseafloor of two contrasting deep‐sea hydrothermal vents. While vent fluids at both Von Damm and Piccard are rich in hydrogen, they differ from one another with respect to pH, methane concentrations, maximum temperature, depth of the vent fields and host rock (German *et al*., 2010; McDermott *et al*., 2015, 2018). Despite these differences and the distinct taxonomic profiles observed by Reveillaud *et al*. (2016) at each vent field, we observed similarities in gene abundance across the 10 Von Damm and four Piccard sites, pointing to metabolic functional redundancy at the community level. These results support previous results from two of these vent sites (Reveillaud *et al*., 2016) and indicate it is a phenomenon robust across multiple vent sites and years. The observation of functional redundancy across Von Damm and Piccard vent fields despite clear taxonomic differences is also consistent with results from DNA analysis of microbial communities in other ecosystems, including the human gut, plant‐associated communities, venting fluids from the Mariana back‐arc, and the cold, oxic subseafloor habitat (Turnbaugh *et al*., 2009; Louca *et al*., 2016, 2018; Tully *et al*., 2018; Trembath‐Reichert *et al*., 2019).

When examining transcript abundance, we observed some large‐scale patterns differentiating microbial communities at Piccard and Von Damm, mainly higher abundance of methyl‐coenzyme M reductase (*mcr*) transcripts and certain hydrogenase transcripts at Von Damm. We also observed a higher diversity of transcripts at certain sites at Von Damm, with Ginger Castle, Hot Cracks and Old Man Tree hosting transcripts across a wider set of genes than at vent sites in Piccard. All of these results are consistent with the fluid chemistry of Von Damm, an ultramafic vent site where methanogenesis is more thermodynamically favourable and with more sources of carbon than at Piccard, resulting in more energy for microbial metabolism and thus more diverse communities and metabolisms (Amend *et al*., 2011; McDermott *et al*., 2015; Reveillaud *et al*., 2016; Anderson *et al*., 2017). The findings show that while many functional genes are equally abundant at the two vent fields, they are selectively expressed based on either local environmental conditions, such as energy availability or fluid flow regimes, or site‐specific biotic interactions, both of which were further explored at higher resolution via MAGs.

We compared the relative abundance of metagenomic reads and transcripts of individual MAGs across vent sites. Through this analysis at the population level, many more differences emerged between Von Damm and Piccard, as well as between individual vent sites within each vent field (Fig. 3). Previous work examining the relative abundance of different taxa based on MAG coverage found differential coverage among MAGs at Piccard and Von Damm (Anderson *et al*., 2017). Our work reveals that samples from Piccard were more similar to each other based on MAG transcript abundance than samples from Von Damm, consistent with 16S rRNA gene analysis (Reveillaud *et al*., 2016). However, many individual vent sites hosted distinct microbial populations, particularly at Von Damm. Like Reveillaud *et al*. (2016), we did not find any significant correlations between geochemical parameters at each site and the gene, transcript, or population abundance patterns, with the exception of Old Man Tree. This site has a high fraction of end‐member source fluid in comparison to all the other vents as measured by magnesium concentration (Supporting Information Table S1). Because there is less seawater mixing with the high temperature end‐member source fluid, a unique and active hyperthermophilic population is sustained at Old Man Tree, distinct from those found at other sites at Von Damm. However, Ginger Castle also hosted a group of high temperature archaea not seen elsewhere at Von Damm, yet the measured geochemical parameters do not explain their presence at only that site and absence at others.

In addition to Old Man Tree, another clear pattern that emerged was the distinction between Shrimp Hole and other vent sites. We observed differences in gene abundance at the community level, as well as differences in microbial population abundance and transcript abundance in both years, suggesting that Shrimp Hole was unique among the vent sites and stable over time. Although the *Methanococci* and *Methanomicrobia* MAGs possess the hallmarks of the methanogenesis cluster described previously, the *Methanomicrobia* MAGs were expressed exclusively at Shrimp Hole, while the *Methanococci* MAGs were exclusive to all other sites. *Methanomicrobia* and *Methanococci* appeared to possess nearly identical sets of pathway capabilities, but phylogenetic trees of these MAGs and a tree of the *mcr* genes within these MAGs both indicated that the *Methanomicrobia* are anaerobic methane oxidizers, while the *Methanococci* are methanogens. This is consistent with 16S rRNA gene surveys and geochemical analyses of vent fluids and animals from Shrimp Hole, which hosts fluids low in hydrogen and methane and displays seep‐like characteristics, including tubeworms with isotopic compositions similar to those found at cold seeps (Bennett *et al*., 2015; McDermott *et al*., 2015; Reveillaud *et al*., 2016). Analysis of *mcr* genes and methanogen MAGs indicated that anaerobic methane oxidation (ANME) appeared to be occurring exclusively at Shrimp Hole in both years, while methanogenesis was occurring elsewhere at Von Damm.

Both the abundance and the activity of microbial populations at each vent site are dictated by a combination of local environmental conditions, such as mixing regime and subseafloor plumbing, as well as biotic interactions with other microbes. While geochemistry and degree of mixing may explain some of the differences seen in the distribution and activity of populations in some sites, it does not explain the restricted distributions of many MAGs to only 1 or 2 vent sites. Our results also suggest there are barriers to exchange between vent sites even within a vent field for some subseafloor microbial populations, resulting in the restricted distribution pattern of particular MAGs. While the exact nature of the barriers to exchange of microbes between sites is unknown at the Mid‐Cayman Rise, work from Axial Seamount, for example, suggests that variations in the local geology at individual vent sites can create fluid flow paths that enable the establishment of stable and distinct microbial communities (Opatkiewicz *et al*., 2009; Akerman *et al*., 2013; Fortunato *et al*., 2018; Stewart *et al*., 2019) much like those observed here.

To look beyond abiotic factors that may influence the observed differential MAG expression patterns, we examined the MAG metabolic pathways. We observed that most functions were conserved within their taxonomic groups, indicating a link between functional potential and taxonomy within these microbial communities. However, we also observed MAGs with similar metabolic potentials but different expression patterns. For example, results here and elsewhere (Reveillaud *et al*., 2016; Anderson *et al*., 2017) showed that the microbial communities at both Piccard and Von Damm encoded high abundances of genes for sulphur oxidation. Different strains from the sulphur‐oxidizing genus *Sulfurovum* were present in varying abundances both within and between sample sites. Previous work showed that Sulfurovum_99 and Sulfurovum_37 are at least 20‐fold more abundant than other *Sulfurovum* MAGs at Piccard, and that these two *Sulfurovum* MAGs were under strong selection at two sites in Piccard (Anderson *et al*., 2017). Based on this new analysis, one possible explanation is that these two *Sulfurovum* strains use different electron acceptors compared to other *Sulfurovum* strains. MCS analysis and manual verification using tblastn revealed that Sulfurovum_99 and Sulfurovum_37 lack c oxidases, unlike other *Sulfurovum* strains (Fig. 4). Moreover, these two *Sulfurovum* lineages have incomplete nitrate reduction pathways: while they can reduce nitrate to nitrite (encoding the nitrate reductases *NR*, *napA*, and *narB*), they cannot further reduce nitrite to ammonia (these MAGs lack the nitrite reductase *nirA*). Nitrate reduction by *nap* has been previously observed to be highly conserved among deep‐sea hydrothermal vent Epsilonbacteraeota (Vetriani *et al*., 2014). Most other *Sulfurovum* strains had the genetic potential to use oxygen and nitrite as electron acceptors, and yet previous work has shown that the two *Sulfurovum* strains apparently lacking these genetic mechanisms appeared to retain a selective advantage over the other Sulfurovum strains (Anderson *et al*., 2017). These *Sulfurovum* MAGs illustrate the non‐conservative nature of certain functions within a clade, which can lead to niche partitioning, different expression levels, and divergent selection pressures within a vent field.

Additional analysis examined cooccurrence patterns and potential metabolic links between different populations. The three combined Nanohaloarchaea and Nanoarchaea MAGs showed MCS values >0.5 for only 3–4 of the 73 pathways that we investigated. The transcript abundances of Nanoarchaea_43 and Nanohaloarchaea_54 are correlated with those of Methanococci_13b and Thiotrichales_3, respectively. These associations, combined with the seemingly sparse metabolic potential of these Nanoarchaea and Nanohaloarchaea, suggests a possible symbiotic relationship, previously observed in Nanoarchaea (Huber *et al*., 2002). The genome of Nanoarchaea_43 is 600 kb in length and is 74.7% complete, and is thus the second smallest genome among the high‐quality MAGs. This is consistent with a genome of reduced metabolic potential. Previous work by St. John *et al*. (2019) showed that a Nanoarchaea MAG recovered from the same metagenome encodes reduced capacity for biosynthesis of amino acids, nucleotides and cofactors, pointing to a symbiotic lifestyle.

It is important to note MCS is a useful but imperfect measure of metabolic potential, since it does not capture how the score is distributed in the genome. For example, a MAG with a score of 0.5 may have the potential for 50% of each step in a pathway (if each step is catalysed by multiple genes), or it may have 100% potential for 50% of the steps in the pathway. Furthermore, using pathway completion data in isolation may miss context‐specific details of a certain module that can reveal more details than its simple presence or absence. For example, in the pathway analysis alone, the methanogenesis and ANME pathways appear identical, requiring a phylogenetic tree to actually differentiate the two. Our analysis was also limited to specific metabolic pathways present in the KEGG metabolic modules database; other pathways may have driven differences in abundance or expression but were not included in our analysis. Finally, it was previously suggested that viruses may drive differences between strains at the Mid‐Cayman Rise (Anderson *et al*., 2017), and viruses have been shown to drive genetic diversity in microbial communities (Weinbauer and Rassoulzadegan, 2003), but this was beyond the scope of the work presented here.

Overall, this study at the Mid‐Cayman Rise provides a compelling example of widespread functional redundancy at the community level, with respect to both gene and transcript abundance. Thus, observations of functional redundancy do not result solely from differences between the fundamental niche (based on gene abundance) and the realized niche (based on transcript abundance). Instead, our results at the genome‐resolved scale suggest that differences in the abundance and expression of specific strains are driven by synergistic differences in the complement of genes within populations, as well as local environmental conditions, including fluid mixing regimes that may impact available energy sources or restrict exchange of organisms between sites. These results highlight the importance of comparing microbial communities at the genome‐resolved scale to better understand microbial community adaptation.

## Experimental procedures

### *Sample collection*

Diffuse flow hydrothermal fluid samples were collected as described in Anderson *et al*. (2017) and Reveillaud *et al*. (2016). Briefly, samples were collected in January 2012 using the Mat sampler on *ROV Jason* aboard the R/V *Atlantis* (FS841‐FS856) (Breier *et al*., 2012). In June 2013, the SUPR sampler was used to collect samples using *HROV Nereus* aboard the R/V *Falkor* (FS866‐FS881) (Breier *et al*., 2009). For all samples, approximately 3–6 l of diffuse flow fluid were pumped through 0.22 μm Sterivex filters (Millipore) on the seafloor for ~30 min while monitoring temperature. Upon vehicle recovery, the filters were flooded with RNALater (Ambion) and sealed with Luer Caps, placed in sterile Falcon tubes at 4°C for 18 h, and stored at −80 °C until extraction.

### *Microbial community DNA and RNA preparation and sequencing*

Total genomic DNA was extracted from half of the Sterivex filter as described in Akerman *et al*. (2013) and Reveillaud *et al*. (2016). RNA was extracted from the other half of the Sterviex filter using the mirVana miRNA isolation kit (Ambion), adding a bead‐beating step using beads from the RNA PowerSoil kit (MoBio) as described in Fortunato and Huber (2016). RNA was treated with DNAse using the Turbo‐DNase kit (Ambion), and then purified and concentrated using the RNAeasy MinElute kit (Qiagen). Metagenomic libraries were prepared as described in Reveillaud *et al*. (2016) and sequenced on an Illumina Hi Seq 1000 at the W.M. Keck Facility in the Josephine Bay Paul Center at the Marine Biological Laboratory. For metatranscriptomic libraries, ribosomal RNA was removed, cDNA synthesized, and libraries prepared using the Ovation Complete Prokaryotic RNA‐Seq DR multiplex system (Nugen) following the manufacturer's instructions. All libraries were sheared to 175 bp using a Covaris S‐series sonicator, yielding paired‐end reads with a 30 bp overlap.

The *iu‐merge‐pairs* script in the illumina‐utils package (Eren *et al*., 2013) was used to merge and filter reads using the –*enforce‐Q30‐check flag*, followed by *iu‐filter‐merged‐reads* in the illumina‐utils package with a maximum mismatch of 2 in the overlap region. This resulted in reads averaging approximately 170 bp in length. Metagenomic reads were assembled using idba‐ud (Peng *et al*., 2012) with default settings. The assemblies from Old Man Tree 2012 (FS841) were excluded from subsequent analysis due to poor assembly.

Metatranscriptomic reads were merged and filtered following the same procedure as for the metagenomic reads. Ribosomal RNA was removed *in silico* by mapping all merged, filtered metatranscriptomic reads to the Silva LSU and SSU Parc databases (release 111) (Pruesse *et al*., 2007; Quast *et al*., 2013) using bowtie2 (Langmead and Salzberg, 2012) with local alignment and default settings, and removing all reads that mapped.

### *Relative abundance of key functional genes in metagenomes and metatranscriptomes*

To determine the relative abundances of genes matching specific metabolic functions, merged, filtered reads were mapped to assembled contigs using bowtie2 (Langmead and Salzberg, 2012) with default settings. We counted the number of reads that mapped to ORFs of defined functions based on annotations with the KEGG Orthology database (Kanehisa and Goto, 2000; Kanehisa *et al*., 2012) according to JGI IMG (Markowitz *et al*., 2012). Manual checking of specific ORFs of interest was conducted with tblastn searches using an e‐value cutoff of 10^−12^. If multiple ORFs had the same annotation, the number of read hits was averaged. Key metabolic genes were defined according to previously published databases, as in Hopkinson and Barbeau, 2012; Reveillaud *et al*., 2016, and Fortunato *et al*., 2018. The normalized gene abundance for each sample was calculated by dividing the number of metagenomic read hits to each key gene by the average number of metagenomic read hits to 35 single copy COGs (Supporting Information Table S7). To calculate the transcript abundance of each gene, the number of metatranscriptomic read hits to each key gene was divided by the total number of metatranscriptomic reads. Again, read hits for ORFs with the same annotation were averaged. Bubble plots were created using the matplotlib Python library (Hunter, 2007).

### *Relative metagenomic and metatranscriptomic abundance of MAGs*

For this analysis, MAGs that had been generated previously from these metagenomes were used (Anderson *et al*., 2017). Briefly, anvi'o (Eren *et al*., 2015) was used to conduct supervised binning of all contigs greater than 1000 bp in length into bins based on tetranucleotide frequency and the relative coverage of each contig across all samples. The completion and redundancy of these bins was estimated using anvi'o, which uses PRODIGAL (Hyatt *et al*., 2010) to call open reading frames and HMMER (Eddy, 2011) to search for and tabulate the occurrence of single‐copy genes for bacteria and archaea from two collections (Campbell *et al*., 2013; Rinke *et al*., 2013, respectively). The taxonomy of bins was identified using PhyloSift (Darling *et al*., 2014). Only metagenomic bins with non‐chimeric taxonomic classification and with completion >70% and redundancy <10% were designated as MAGs and used for subsequent analysis. The normalized coverage was calculated by dividing the average coverage across the MAG by the number of reads in the metagenome. To calculate the relative transcript abundance of each MAG, the average coverage of metatranscriptomic reads for each MAG was divided by the total number of metatranscriptomic reads at the sample where MAG transcript abundance is measured. Heatmaps were created using the Seaborn Python visualization library in matplotlib (Hunter, 2007), and hierarchical clustering was performed with the seaborn *clustermap* method using Euclidean distance and Ward linkage.

### *MCS calculations*

The module completion ratio or MCS reflects the functional potential of MAGs by quantifying the relative number of genes from a specific metabolic pathway that are present within a MAG (Takami *et al*., 2012). MCS is a real number between 0 and 1 and is calculated as the proportion of KEGG orthologs possessed by the MAG for each reaction step within a metabolic module. ORF calls and annotations from the JGI IMG pipeline were used to calculate the MCS. The completeness proportions (also between 0 and 1) for all reactions in the module were then averaged to obtain a single MCS for a MAG for a specific module. Heatmaps were created using the Seaborn Python visualization library in matplotlib, and hierarchical clustering was performed with the seaborn *clustermap* method using Euclidean distance and Ward linkage.

To determine the relative enrichment of specific MCSs in MAGs at Piccard versus Von Damm, a two‐tailed Mann–Whitney U test was used as implemented in R (R Core Team, 2013) using *wilcox. test()*, with the null hypothesis that two samples being compared have the same distribution of values.

### *Methanogen and ANME gene identification and phylogenetic trees*

To search for evidence of methanogenesis and ANME potential, genes encoding *mcrA* (KO number K00399) were identified in all metagenomic contigs based on KEGG annotations through the JGI IMG pipeline. The following reference mcrA sequences were identified in the NCBI Protein database: ANME mcrA uncultured archaeon (AAQ63481.1), ANME mcrA uncultured archaeon (AAQ63476.1), ANME mcrA ANME‐2 cluster archaeon HR1 (PPA79495.1), ANME mcrA uncultured archaeon (CBH39484.1), ANME‐1 mcrA (AFS68368.1), and ANME‐1 mcrA (ADW65684.1). mcrA sequences were also identified in the following genomes on the NCBI Genomes database: *Methanothermobacter marburgensis* str. Marburg (MTBMA_RS07375), *Methanosarcina barkeri* strain Fusaro (MBAR_RS04805), *Methanothermobacter thermautotrophicus* strain ATCC 29095 (MTH_RS05360), *Methanopyrus kandleri* strain AV19 (Q49605.2), *Methanococcus voltae* AE (MVOL_RS05625), *Methanothermus fervidus* DSM 2088 (MFER_RS03735), *Methanococcus vannielii* SB (MEVAN_RS04455), *Methanocaldococcus jannaschii* strain ATCC 43067 (MJ_RS04540), *Methanococcus maripaludis* S2 (MMP_RS08025), *Methanobrevibacter ruminantium* strain ATCC 35063 (WP_012956722.1), *Methanocella arvoryzae* strain DSM 22066 (RCI_RS05310), *Methanococcoides burtonii* strain DSM 6242 (MBUR_RS12560), *Methanothrix soehngenii* strain ATCC 5969 (WP_013718622.1), and *Methanoculleus bourgensis* strain ATCC 43281 (CCJ36661.1). To make a phylogenetic tree of *mcrA* genes, amino acid alignments were generated using muscle v3.8.31 (Edgar, 2004) and maximum likelihood trees were generated with 100 bootstraps using RAxML version 8.2.9 (Stamatakis, 2014) using the 'rapid bootstrap' method and the 'PROTGAMMAAUTO' option to model rate heterogeneity.

To make a phylogenetic tree of methanogen and ANME MAGs and reference genomes, we identified the putative taxonomy of MAGs and identified single copy universal genes using PhyloSift (Darling *et al*., 2014) using 'phylosift all' with the 'isolate' and 'besthit' flags. We included the following reference genomes from the NCBI Genomes database:

*Methanobrevibacter woesi* strain DSM 11979 (MZGU01000001.1), *Methanocaldococcus jannaschii* DSM 2661 (L77117.1), *Methanococcus maripaludis* C7 (CP000745.1), *Methanococcus voltae* A3 (CP002057.1), *Methanocorpusculum parvum* strain XII (LMVO01000001.1), *Methanomicrobia* archaeon JdFR‐19 JGI24020J35080_1000033 (MTMG01000017.1), *Methanomicrobiaceae* archaeon UBA2 (DAQH01000006.1), *Methanopyrus kandleri* AV19 (AE009439.1), *Methanosarcina barkeri* MS (CP009528.1), *Methanosphaerula palustris* E1‐9c (CP001338.1), *Methanothermobacter thermautotrophicus* str. Delta H (AE000666.1), *Methanothermococcus okinawensis* IH1 (CP002792.1), and *Thermococcus barossii strain SHCK‐94* (CP015101.1). We used PhyloSift to identify single copy universal genes. The concatenated protein alignments generated by PhyloSift were used to create phylogenetic trees using RAxML using the same method as above. Trees were visualized using the Interactive Tree of Life website (http://www.itol.embl.edu) (Letunic and Bork, 2011).

## Data Availability Statement

Metagenomic and metatranscriptomic reads are deposited under study accession code PRJEB15541 in the EMBL‐EBI European Nucleotide Archive (ENA) database. All code used for this project has been deposited to GitHub at https://github.com/carleton-spacehogs/functional_metagenomics.

## Supporting information


**Supplementary Table 1**. Temperature, cell count, pH, and geochemistry data associated with each of the samples analysed for this study. No MT, no metatranscriptome was sequenced for this sample. B.d., below detection levels.
**Supplementary Table 2**. Information about all MAGs analysed in this study.
**Supplementary Table 3**. KEGG module accession numbers.
**Supplementary Table 4**. Modules that were significantly enriched according to MCS at Piccard relative to other vent fields or sites.
**Supplementary Table 5**. Modules that were significantly enriched according to MCS at Shrimp Hole relative to other vent fields or sites.
**Supplementary Table 6**. Modules that were significantly enriched according to MCS at Von Damm and Shrimp Hole relative to Piccard.
**Supplementary Table 7**. List of single‐copy COGs used for normalization of gene abundances based on metagenomic mappings.
